# Photoreceptor replacement: a disease-agnostic approach for the treatment of advanced retinal degeneration

**DOI:** 10.1038/s41433-026-04742-4

**Published:** 2026-07-25

**Authors:** Rachael A. Pearson

**Affiliations:** Ocular Cell and Gene Therapy Group, Centre for Gene Therapy and Regenerative Medicine, King’s College London, Guy’s Hospital, London, United Kingdom

**Keywords:** Diseases of the nervous system, Visual system

## Abstract

Retinal dystrophies such as retinitis pigmentosa (RP) and age-related macular degeneration (AMD) affect millions of individuals worldwide and remain leading causes of irreversible blindness. Photoreceptor transplantation has emerged as a promising and disease-agnostic therapeutic approach for restoring vision in advanced degenerative retinal diseases characterised by the loss of rods and/or cones. This brief review summarises some key areas of progress and future directions for photoreceptor replacement therapy, with consideration given to translating pre-clinical findings to the clinic.

## Background: rationale for photoreceptor cell replacement

The retina is a highly organised, laminated neural tissue. Photoreceptors are situated in the outer nuclear layer (ONL) and form synaptic connections with bipolar cells (BCs) and horizontal cells, located in the inner nuclear layer (INL). These, in turn, connect to retinal ganglion cells (RGCs), which transmit visual information to the brain. A photoreceptor’s location in the retina dictates its contribution to the visual system’s representation of the world around us. While most patients presenting in clinic already have substantial photoreceptor loss, many of the inner retinal neurons survive even very late in degeneration [1–3]; as photoreceptors are afferent neurons, transplanted cells would, theoretically, need to make only a single synaptic connection to recipient BCs to restore the visual circuit. This, together with recent developments in the ability to generate photoreceptors from renewable sources like embryonic stem cells (ESCs) or induced pluripotential stem cells (iPSCs), presents an opportunity to reverse sight-loss by replacing the lost photoreceptors with healthy ones. Restoring functional neuronal connectivity in any advanced disease by transplantation is ambitious. However, the fovea and surrounding macula are crucial for high-acuity daylight vision yet occupy a small area and cone photoreceptors are highly concentrated in these regions; the fovea is ~1.8 mm^2^ with ~200,000 cones/mm^2^ making targeted, focal repair a plausible goal. Key to this strategy’s success is the ability of donor photoreceptors to mature in situ and for both donor photoreceptors and host inner retinal neurons to exhibit neuronal plasticity and form new synaptic connections. For complex conditions such as AMD, it will likely be necessary to also replace the RPE, but successful photoreceptor replacement is central to all these approaches.

## Early research

Pioneering studies explored photoreceptor cell transplantation using one of two delivery approaches; transplantation of pieces of intact, typically foetal, retina or cells delivered as a single cell suspension, into adult degenerating (usually rodent) retinas. The former approach brings the benefits of a pre-organised tissue but involves invasive surgery and the introduction of a second inner retina (see later for further discussion), while the cell suspension approach offers surgical simplicity and the ability to sort cells before transplant. However, this comes at the expense of organisation within the graft itself (Table 1).

**Table 1:** Potential advantages and disadvantages of current cell suspension and retinal sheet transplantation approaches.

Delivery method	Advantages	Disadvantages
Cell suspension	Option to enrich for photoreceptors and remove contaminants	Sorting increases manufacturing complexity
	Cells delivered to subretinal space and in direct contact with target host inner retina	Variable polarisation/organisation of grafted cells
	Simple, minimally invasive procedure	Potential for reflux
	Adjustable dosing	
	Low immunogenicity (if sorted)	
	Evidence of survival >6 months	
	Evidence of synaptic connectivity and functional rescue	
Retinal patches	Potential preservation of graft lamination	Orientation issues and formation of rosettes
	Patches delivered to subretinal space and in direct contact with target host inner retina	Mixed cell population including cells that may limited donor-host synaptic connectivity
	Low immunogenicity	Surgical complexity and invasive procedure
	Evidence of survival >6 months	Limited area/dosing
	Evidence of synaptic connectivity and functional rescue	

A variety of cell types were initially trialled, including various immature and adult stem cell populations, as well as retinal progenitor cells. Indeed, mixed populations of human foetal retinal cells were transplanted in clinical trials as early as 1999 [4]. While these studies demonstrated cell survival, evidence of correct maturation and functional integration was limited [4–7]. Nonetheless, they established the feasibility of introducing new photoreceptors into a diseased retina. In 2006, again using murine donor cells and recipients, we demonstrated that donor cells taken from a specific window in their development enabled their survival and subsequent differentiation into mature photoreceptors; specifically, cells that were committed to a photoreceptor fate but had not yet fully matured, so-termed post-mitotic photoreceptor precursors, performed better than more immature retinal progenitor/precursor cells or older, fully differentiated photoreceptors [8]. Follow-on studies using rod [9–11] or cone [12] post-mitotic precursors yielded similar findings. If present in sufficiently large numbers, these could also rescue retinal function and visually evoked behaviours [12, 13].

## Stem cell–derived photoreceptors

ESC and iPSC technologies transformed the prospects for cell replacement therapies, including photoreceptor transplantation, offering a potentially inexhaustible, scalable supply of donor material [14–16]. The challenge, however, has been to generate stage-specific cells that fully recapitulate photoreceptor development, with many early standard 2D adherent culture protocols yielding partial, but incomplete, differentiation. In 2011, Sasai and colleagues demonstrated a self-organising “3D” retinal organoid derived from murine ESCs that recapitulated many of the hallmarks of retinal development [17]. We, and others, have subsequently developed a variety of related approaches and protocols to achieve robust production of rod and cone photoreceptors in vitro [18–22]. These variously use a combination of 2D adherent and/or 3D free-floating steps, and sequential application of exogenous factors designed to specify neural, retinal and ultimately photoreceptor fate specification; the reader is directed to O’Hara and Gonzalez-Cordero [23] for a comprehensive review on this topic. Utilising a combined 2D/3D method, we successfully generated stage-specific rods and cones from murine ESCs that when transplanted performed in a manner equivalent to donor-derived post-natal mouse photoreceptors [22]. With the aim of targeting macula diseases, we sought to further adapt these protocols to generate large numbers of cone photoreceptors for transplantation, successfully achieving this with both mouse [24] and human ESCs [21]. Donor cells (for example, rod or cone precursors) can be selectively enriched for research purposes using viral vectors driving cell- and differentiation stage-specific fluorescent reporter expression [21, 24, 25] or cell surface markers [11, 26–28], combined with FACs or MACS-based sorting techniques, the latter being gentler and generally offering better cell survival [10, 11, 29].

## Material transfer vs. integration

The majority of earlier photoreceptor transplantation studies utilised transgenic fluorescent reporters (e.g. *Nrl.Gfp, Crx.Gfp* [8, 11, 12, 30]) and were performed into models of partial degeneration [8–11, 13, 31], where some of the host photoreceptors remained, the rationale being that this would provide some degree of cytoarchitecture for the transplanted cells to integrate within. For many years, it had been understood that the transplanted photoreceptors migrated into and structurally integrated within the remaining ONL of the host retina and formed synaptic connections. However, in 2016 in a transformative series of studies, we, and others, demonstrated that many earlier observations were misinterpreted: rather than integrating *within* the remaining host ONL, many donor photoreceptors were instead transferring cytoplasmic material, including both phototransduction-related proteins and the genetically-encoded fluorescent reporters, to the remaining host photoreceptors cells [32–36]. This rendered the host cells functional [13] but also led to them appearing donor-derived when they were not. This discovery dramatically reshaped our interpretation of prior results [37], emphasising the need for even more rigorous lineage-tracing, genetic barcoding, and quantitative analysis to distinguish structural integration from molecular exchange [33, 38]. Careful genetic tracing and real-time imaging showed that true integration does occur in the intact host retina, albeit at lower levels than previously thought [32, 33, 36, 39]. Subsequently, we and the Wallace group independently identified the cellular mechanism by which material transfer occurs, which is by the formation of nanotube-like membranous bridges that form a conduit between donor and host photoreceptors [40, 41]; this cytoplasmic bridge between cells results in the robust exchange of structural and cytoplasmic proteins at a level sufficient to restore function in the accepting cells and bring about visually-evoked behaviours in murine models of partial photoreceptor degeneration [13, 32]. Interestingly, this exchange appears to be specific between photoreceptors of the same species (mouse to mouse, human to human) but does not occur in xenotransplantations [21, 39, 41].

## Functional integration in advanced disease

**Fig. 1:**
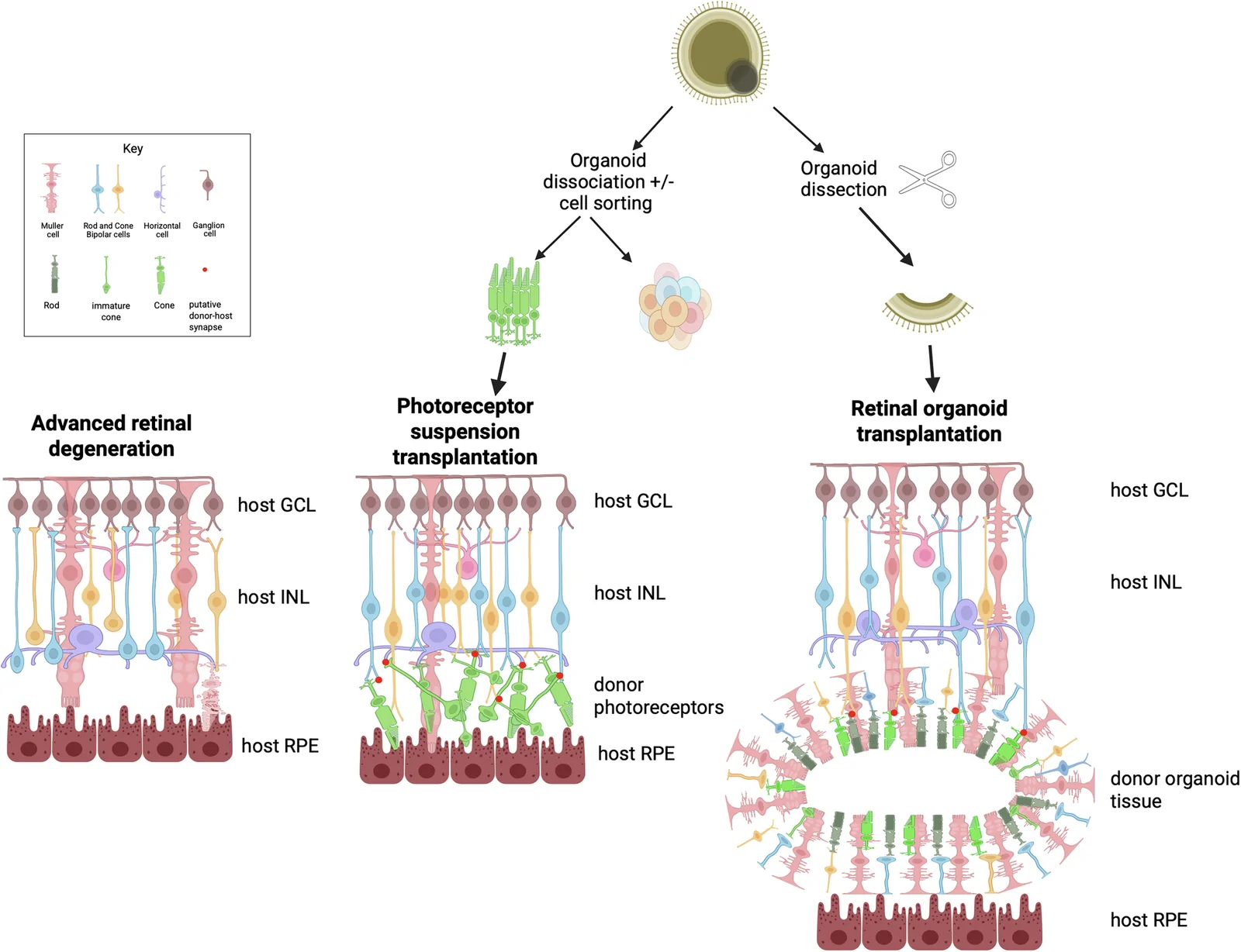
Schematic illustrating the cell suspension and organoid graft approaches to photoreceptor replacement in advanced retinal degeneration. ESC or iPSC-derived retinal organoids provide donor tissue for retinal patches or are dissociated and sorted using cell-specific markers to enrich for photoreceptors (rods & cones, or cones-only) or their precursors prior to transplantation. Transplants of purified cone photoreceptors (*green*) have been shown to make putative synapses (*red dots*) with host bipolar cells (*blue, yellow*), as have cells within retinal organoid sheets. Note that organoid sheets can often form rosettes. Both approaches have yielded improvements in retinal behavioural function after transplantation.

In the intervening years, there has been renewed focus on the arguably more clinically relevant models of advanced degeneration (Fig. 1), where the absence of most/all host photoreceptors largely remove the confounding complications in interpretation that may arise from material transfer. Many earlier studies examining more advanced disease explored the use of sheets of human fetal [42–45] or rodent [46] retina, and, more recently, pieces cut from mouse [47, 48] or human [49–53] PSC-derived retinal organoids. These studies have yielded some promising indications of improved function. To date, rods have made up the majority of the photoreceptors in these transplanted grafts; regarding macula-directed therapies, while cone-rich organoids have been generated [54, 55], they have not been trialled for transplantation. Moreover, the inclusion of an unknown quantity and identity of inner retinal neurons within the grafted tissue limits the capacity for widespread donor photoreceptor-host BC contact [56]. Since not all of these studies used models/stages of degeneration where photoreceptor loss is complete, it is also not clear whether the reported improvements in function result from synaptic connectivity between donor photoreceptors and host BCs or instead reflect activity arising within the graft itself, or indeed a rescue of residual photoreceptor and/or other retinal neuronal function, due to neurotrophic effects or material transfer to any remaining host photoreceptors [57]. Indeed, an important limitation of many studies (both cell suspension and sheet) has been the lack of appropriate controls for potential trophic and material transfer effects.

Demonstrating synaptic connectivity between donor and host is challenging. Standard methods of analysis, using immunolabelling for pre- and post-synaptic proteins or electron microscopy, are complicated by the lack of organisation within the grafted area; BCs downregulate the expression of post-synaptic receptors, like mGluR6, and retract their dendrites during degeneration so both donor and host must re-elaborate axonal and dendritic projections to reconnect. As such, putative synaptic contacts typically occur throughout the area of transplant, rather than in the highly ordered confines of the OPL, as occurs in the intact retina. Thus, most conclusions around synapse formation have been necessarily extrapolated from assessments of proximity between fluorescent puncta ostensibly labelling pre- and post-synaptic proteins [52]. Whilst not definitive, numerous studies have found pre- and post-synaptic proteins in very close proximity, indicative of the formation of synaptic-like contacts [52, 58–63]. Regarding organoid-derived patches, Mandai and colleagues have made very significant progress in recent years towards achieving bone fide donor-host connectivity, by genetically modifying the donor tissue to prevent the production of BCs [58, 64, 65]. This yields retinal tissue with a substantially reduced inner retina cell contribution, enabling significantly improved donor/host interactions with evidence of synaptic connectivity and functional improvements [52, 64, 65].

An indication that human photoreceptors transplanted as cell suspension may make new connections came from a study by Duebel and colleagues [66], who generated optogenetically-engineered human iPSC-derived photoreceptors and recorded a modest improvement in retinal function in response to very bright light in the *Cpfl1/Rho*^−/−^ and *Rd1* models of advanced retinal degeneration. Notably, however, the control was normal iPSC-derived cones, which failed to yield any response. We therefore sought to determine whether enriched populations of human cones could rescue retinal and/or visual function. Using two different models of end stage disease, the *Rd1* model, and the *Aipl1*^*−/−*^ model of Leber congenital amaurosis, we showed that hESC-derived cone photoreceptors can repopulate an area of ~5 mm^2^ and restore both retinal function and visually evoked behaviours [59, 60, 67]. Immunohistochemistry showed re-expression of the post-synaptic receptor, mGluR6, and this was frequently seen close to the pre-synaptic proteins, Ribeye and PSD-95. Electrophysiological recordings of RGCs showed that transplanted human cones detect light and drive inner retinal activity, displaying an array of responses to simple light-on/light-off stimuli within a physiological relevant range of intensities, while improvements in optokinetic head tracking show that information is received by other visual areas. Human cones derived from a patient with achromatopsia (i.e. non-functional) served as a control for neurotrophic and materials transfer effects and show no improvements in either retinal or behavioural function [59]. *Aipl1*^*−/−*^ presented a particularly extreme case for rescue since degeneration begins before photoreceptor-BC synapses establish in development [68]. Since AMD is a key target condition for photoreceptor transplantation, likely in conjunction with retinal pigment epithelium (RPE) replacement, we have since extended these findings to successfully rescue aged (>1 yr old) *rd1* mice [67], with rescue lasting >8-months without a noticeable decline in responses. Together, these provide increasing support for the potential of transplanted human photoreceptors, delivered either as enriched photoreceptor cell suspensions or as engineered photoreceptor-rich patches, to make new synaptic connections with the host retina and bring about meaningful functional improvements (Fig. 1).

## Future challenges for improving rescue

Achieving high efficiency, correctly organised donor-host connectivity is vital for restoring useful vision, and photoreceptor replacement therapy must achieve this amidst ongoing degeneration. In many degenerations, the remaining inner retinal neurons continue to remodel, and even die, long after the photoreceptors have died [1, 2, 69, 70] and reactive gliosis, while variable between diseases [71, 72], can eventually entomb the remaining cells, raising concerns for the feasibility of photoreceptor transplantation in advanced disease [73, 74]. In AMD, the RPE is also affected and effective treatments for this condition will need to address its replacement alongside photoreceptors. RPE transplantation and RPE/photoreceptor co-transplantation strategies are beyond the scope of this brief review, but are reviewed elsewhere [75, 76].

We, and others, have sought to define the factors that influence photoreceptor transplantation success, for example showing that glial scarring may be less of an issue than first anticipated [74, 77], with reactive Müller Glia remodelling to incorporate the transplanted cells, in at least three different models of progressive or advanced disease [59–61]. Also surprising is that, as noted above and despite widespread dendrite retraction and downregulation of post-synaptic receptor expression, the surviving BCs in the recipient retina exhibit remarkable plasticity and re-elaborate dendrites and re-express mGluR6 in response to transplantation of photoreceptors and retinal grafts [59, 61, 64, 67]. This was not a given since, while many inner retinal neurons survive long after complete photoreceptor loss, significant structural and functional changes still occur, including changes in post-synaptic receptor expression, extensive dendritic and axonal remodelling, and even cell death [1, 2, 73, 78, 79]. Histological evidence also supports the involvement of the lateral pathways in the restored connectome: we have found that host-derived Calbindin^+ve^ horizontal cells (HCs) re-form their projections throughout the OPL in response to human cones [60, 67], and Mandai and colleagues similarly reported the involvement of graft and host HCs in the formation of synapses between graft retinal tissue and the host retina [62]. However, we find PKCα^+ve^ ON (rod &/or cone) BCs respond more vigorously than Secretagogin^+ve^ ON or OFF cone BCs, extending their dendrites into the donor cell mass [60], resembling immature BCs during OPL formation [80]. These differences are notable; recent structural analysis of laser-induced scotomas in rabbits and NHPs showed differences in how rod ON and cone ON and OFF BCs sought out new connections with healthy photoreceptors at the scotoma edge, and how well they retained synaptic connections with RGCs [69, 81], indicating different changes between ON and OFF pathways across degeneration. Such structural changes may be reflected functionally in the transplanted host retina: we found spike-sorting of RGC responses from human cone-transplanted retinas in response to full-field light flash stimuli revealed a predominance of ON, compared to ON-OFF and OFF, responses [59, 60], with Lako and colleagues reporting similar findings from photoreceptor precursor cells [82]. A similar profile was seen by Mandai and colleagues after transplantation of retinal organoid-derived patches [48, 64, 65]. Interestingly, direct stimulation of ON BCs within the *Rd1* retina using the optogenetic actuator ReaChR revealed responses broadly similar to wildtype in terms of diversity and complexity [83], suggesting that there is potential to restore complex retinal processing after photoreceptor transplantation, even if this predominantly relies on recruitment of the ON system. However, further research is required to probe retinal processing post-transplantation and characterise the extent of transplant-restored vision in a systematic manner.

Photoreceptors are highly polarised cells, with inner/outer segments projecting apically to physically interact with the RPE and axons projecting basally to synapse with BCs and HCs. This organisation is lost during degeneration and is difficult to restore in transplantation. Cell suspension approaches are particularly challenging as dissociation causes the transplanted donor cells to lose projections and exhibit a rounded morphology; they must then elaborate new axonal projections and segments and do so in a fully mature environment without the normal developmental cues that guide these processes. Organoid patches also present some challenges regarding polarity; achieving correct orientation during surgery is not straightforward. Moreover, many are transplanted whilst immature and many go on to develop rosettes, with photoreceptor inner/outer segments projecting inwards into the rosette centre, rather than apically towards the RPE [48, 50, 56] (Fig. 1). Both approaches will likely benefit from future developments that help to promote polarisation within the graft. Re-introduction of developmentally-relevant growth factors, such as IGF-1 [84] were trialled early on, but have not been further developed since the discovery of material transfer complicated their interpretation [32]; however, with new strong evidence to support synaptic integration of both cone photoreceptor-only [59, 60] and organoid-derived grafts [52, 64], such approaches are perhaps now worth revisiting.

## Clinical translation – GMP manufacturing

The past decade has seen significant progress and an increasingly robust body of evidence to support photoreceptor replacement as a therapeutic approach to restore sight loss, justifying efforts on how to translate this to the clinic.

### GMP manufacturing requirements

GMP manufacturing requires careful consideration of the methodologies used to produce cell products for clinical application. It typically requires the use of Xeno-free, chemically defined, and GMP-certified media and reagents and the cells must undergo extensive characterisation, including identity testing, sterility, mycoplasma testing, viral screening, karyotype stability, and assessments for pluripotency and differentiation capacity. Below, I consider some of the aspects of GMP processing that are particularly pertinent to photoreceptor-based products.

### Differentiation and expansion under GMP

The differentiation process, from pluripotent cells to retinal organoids or photoreceptor-rich populations, must be standardised, reproducible, and validated. A key challenge for human retinal cell-based products is the long duration of organoid differentiation, which follows the same time-course of human development [21]; to our knowledge, there is no published evidence of successful restoration of function in advanced disease (where neuroprotection/materials transfer can be excluded) using methods of expedited differentiation. Other considerations include stability of lineage commitment, batch-to-batch consistency and scalability for later stage development. The long differentiation timeframes mean that the use of closed or semi-closed culture systems is essential wherever feasible to reduce contamination risk.

### Cell isolation and purification

The cell suspension approach often involves sorting mixed populations to enrich for stage-specific photoreceptor precursors and remove non-photoreceptor cell types. Preclinical methods such as the use of viral vectors and fluorescent reporters are not compatible with GMP manufacturing. However, post-mitotic photoreceptor precursors also express cell surface markers, which can be used to selectively enrich these cells from a heterogenous starting populations [27, 28, 85, 86] and GMP compliant cell sorting is now available, making this feasible for clinical grade manufacturing. The interest in cell therapies has also brought new label-free methods of sorting to market, which exploit biophysical properties like size, stiffness and viscosity. Such features have been successfully employed to isolate rod photoreceptors from mixed cell suspensions [87, 88].

Cell sorting also brings potential advantages in terms of safety, as heterogeneous cell populations increase risks of tumorigenesis and immunogenicity. While the risk of including undifferentiated stem cells is low given the lengthy differentiation times employed in many protocols, other cell types like Müller glia and RPE retain the capacity for proliferation even after terminal differentiation [89], introducing another variable to treatment outcomes. Regarding immunogenicity, early safety studies in humans using ESC-derived RPE [89], foetal neural retina [4, 43] and PSC-derived retinal organoid sheets [90] all suggest that non-autologous grafts are tolerated, with retinal tissue exhibiting particularly low immunogenicity [91]. Since photoreceptors are non-antigen presenting (in contrast to RPE, for example), purified populations of photoreceptors may be even less likely to trigger the host immune system, although further studies are required to test this directly.

### Cryopreservation

Published data demonstrating functional rescue following the transplantation of cell suspensions or organoid sheets has been achieved using fresh tissue or cells. However, sterility testing, an essential part of Quality Control, presents challenges for a living therapeutic product due to the long incubation times involved, during which time the product requires on-going tissue culture. Moreover, cryopreservation is a key enabling technology in regenerative medicine, providing stable and secure extended storage and transportation of the final product. Neuronal populations are notoriously difficult to cryopreserve, often exhibiting low viability and functional loss, and retinal tissue and photoreceptors are no exception. While there are reports of cryopreservation and subsequent recovery and culture of immature retinal organoid tissue (<d50) and CD73+ rod precursors [85, 92], evidence of successful functional recovery after transplantation using cryo-recovered tissue in the published literature is lacking. Gagliardi and colleagues reported being able to transplant cryo-recovered photoreceptors but only after a period of recovery culture [85], which is not practical within a GMP framework. Recovery of intact retinal tissue appears to be particularly challenging for intact tissue grafts; Watari and colleagues note that cryo-recovered retinal tissue showed significantly impaired engraftment compared to fresh tissue [93].

### Potency assays

Potency assays represent a challenging aspect of generating a GMP-compliant manufacturing strategy for photoreceptor therapies. Functional integration cannot be readily tested in vitro, so surrogate assays, such as quantification of gene expression for photoreceptor markers and morphological assessments, are required. However, regulatory bodies typically require these to correlate with expected in vivo performance.

### Safety and regulatory considerations

Safety concerns unique to cell/tissue therapies include tumorigenicity, immunogenicity, ectopic tissue formation, and potential for unwanted cell types in the final transplant. As discussed above, photoreceptor-based products are unlikely to contain many/any undifferentiated stem cells, but the proliferative and immunogenic potential of other retinal cells must be considered in mixed population transplants. If using allogeneic cells, immunosuppression regimens and HLA-matched banks may be employed. Meanwhile, the FDA and EMA classify photoreceptor and retinal tissue transplants as advanced therapy medicinal products (ATMPs), requiring detailed data on manufacturing processes, preclinical toxicology, biodistribution, and long-term safety prior to clinical trial approval.

## Outlook

Photoreceptor transplantation is advancing rapidly, with academic groups and companies beginning to establish GMP manufacturing methods of fresh retinal organoid and/or photoreceptors [92, 94] and progressing toward or entering early-phase clinical trials [95]. Success will depend on further biological insights from pre-clinical studies, particularly around understanding the accuracy of the repaired connectome and improving the efficacy of donor-host connectivity, refinement of approach obtained through clinical trials, and the robustness, scalability and cost-effectiveness of GMP manufacturing processes. Together, these will enable photoreceptor transplantation to become a realistic therapeutic option for the many individuals affected by advanced retinal degeneration.
